# Prognostic utility of lipoprotein(a) combined with fibrinogen in patients with stable coronary artery disease: a prospective, large cohort study

**DOI:** 10.1186/s12967-020-02546-y

**Published:** 2020-10-01

**Authors:** Yan Zhang, Jing-Lu Jin, Ye-Xuan Cao, Hui-Hui Liu, Hui-Wen Zhang, Yuan-Lin Guo, Na-Qiong Wu, Ying Gao, Qi Hua, Yan-Fang Li, Rui-Xia Xu, Chuan-Jue Cui, Geng Liu, Qian Dong, Jing Sun, Jian-Jun Li

**Affiliations:** 1 Division of Dyslipidemia, State Key Laboratory of Cardiovascular Disease, FuWai Hospital, National Center for Cardiovascular Diseases, Chinese Academy of Medical Sciences, Peking Union Medical College, BeiLiShi Road 167, Beijing, 100037 China; 2 Department of Cardiology, Xuanwu Hospital, Capital Medical University, Beijing, China; 3 Department of Cardiology, Beijing Anzhen Hospital, Capital Medical University, Beijing, China

**Keywords:** Lp(a), Fibrinogen, CAD, CVEs

## Abstract

**Background:**

Elevated lipoprotein(a) [Lp(a)] and fibrinogen (Fib) are both associated with coronary artery disease (CAD). The atherogenicity of Lp(a) can be partly due to the potentially antifibrinolytic categories. We hypothesize that patients with higher Lp(a) and Fib may have worse outcomes.

**Methods:**

In this prospective study, we consecutively enrolled 8,417 Chinese patients with stable CAD from March 2011 to March 2017. All subjects were divided into 9 groups according to Lp(a) (Lp(a)-Low, Lp(a)-Medium, Lp(a)-High) and Fib levels (Fib-Low, Fib-Medium, Fib-High) and followed up for CVEs, including nonfatal acute myocardial infarction, stroke, and cardiovascular mortality. Kaplan–Meier, Cox regression and C-statistic analyses were performed.

**Results:**

During a median of 37.1 months’ follow-up, 395 (4.7%) CVEs occurred. The occurrence of CVEs increased by Lp(a) (3.5 vs. 5.3 vs. 5.6%, *p* = 0.001) and Fib (4.0 vs. 4.4 vs. 6.1%, *p* < 0.001) categories. When further classified into 9 groups by Lp(a) and Fib levels, the CVEs were highest in the 9th (Lp(a)-High and Fib-High) compared with the 1st (Lp(a)-Low and Fib-Low) group (7.2 vs. 3.3%, *p* < 0.001). The highest risk of subsequent CVEs was found in the 9^th^ group (HR_adjusted_ 2.656, 95% CI 1.628–4.333, *p* < 0.001), which was more significant than Lp(a)-High (HR_adjusted_ 1.786, 95% CI 1.315–2.426, *p* < 0.001) or Fib-High (HR_adjusted_ 1.558, 95% CI 1.162–2.089, *p* = 0.003) group. Moreover, adding the combined Lp(a) and Fib increased the C-statistic by 0.013.

**Conclusion:**

Combining Fib and Lp(a) enhance the prognostic value for incident CVEs beyond Lp(a) or Fib alone.

## Background

Despite significant advances in the diagnosis and therapy of cardiovascular disease (CVD), patients with established coronary artery disease (CAD) are generally at higher risk of developing recurrent cardiovascular events (CVEs) than the primary prevention individuals [1]. Clinical trials revealed that in the short time window only 20–30% of patients benefit even if traditional risk factors were well managed [2, 3]. As a result, identifying additional modifiable risk factors is necessary to further improve CVEs prediction in the management of patients with established CAD.

Evidence have established high lipoprotein(a) (Lp[a]) levels are associated with high risk of CVD, observationally and causally from human genetics [4–6]. Multiple studies have indicated that high Lp(a) cause CVD in a primary prevention setting, moreover, Lp(a)-lowering by 50 mg/dL may reduce CVD by 20% in a secondary prevention setting [7]. AS well known, Lp(a) is composed of an LDL-like particle in which apoB is covalently bound by a single disulfide bond to apolipoprotein(a) (apo[a]). Therefore, the pathogenic role of Lp(a) was supposed to be involved in atherosclerosis and thrombosis formation [8]. In fact, the recent study implied that the mortality effect of high lipoprotein(a) is above that explained by its cholesterol content but the number of KIV-2 repeats in the apo(a) [9]. Originally, apo(a) has evolved from the plasminogen gene through duplication and remodeling. Unlike apolipoprotein B, apo(a) does not contain lipid domains or transport lipid, but instead, it potentiates atherothrombosis through additional pathways including proinflammatory, and potentially antifibrinolytic effects by inhibiting plasminogen activation [10]. As one of the important components of fibrinolytic system, plasma fibrinogen (Fib) has been proved to be a pivotal CVD risk factor [11–13]. However, little is known about the inter-relationship of Lp(a) and Fib in the CVEs risk prediction in the secondary prevention setting.

As a consequence, we hypothesize that there is a risk interaction between Lp(a) and Fib, and patients with high Lp(a) and Fib may have worse outcomes. We thereby sought to investigate the association of Lp(a) and Fib in predicting CVEs in patients with stable CAD (SCAD) in the current study.

## Methods

### Study population

Our study complied with the Declaration of Helsinki and was approved by the hospital’s ethics review board (Fu Wai Hospital, National Center for Cardiovascular Diseases). Informed written consents were collected from all patients obtained in this study.

From March 2011 to March 2017, a total of 10,042 Chinese patients with clinical symptoms such as angina pectoris, or chest distress were recruited in our study. The inclusion criteria were patients with stable and angiography-proven CAD (coronary stenosis ≥ 50% of at least one coronary artery). The exclusion criteria were as follows: (1) acute coronary syndrome (ACS); (2) previous myocardial infarction (MI), previous percutaneous coronary artery intervention or bypass grafting; (3) heart failure; (4) other disease status such as severe liver and/or renal insufficiency, thyroid dysfunction, systematic inflammatory disease, and malignant disease. Therefore, 8417 patients were finally enrolled in the current analysis.

Patients were followed up at 6 months intervals by means of direct interview or telephone. The follow-up was performed by trained nurses or physicians who were blinded to the clinical data. The primary end points were cardiovascular mortality, nonfatal MI, and stroke. Nonfatal MI including ST-segment–elevation MI and non–ST-segment–elevation MI was diagnosed as positive cardiac troponins along with typical chest pain or typical electrocardiogram serial changes. Stroke was confirmed by specialist physicians according to the presence of typical symptoms and imaging.

Diabetes mellitus (DM) was diagnosed by fasting plasma glucose ≥ 7.0 mmol/L, the 2 h plasma glucose of the oral glucose tolerance test ≥ 11.1 mmol/L, or current use of hypoglycemic drugs or insulin. Hypertension was defined as self-reported, currently taking antihypertensive drugs, or recorded systolic blood pressure ≥ 140 mmHg or diastolic blood pressure ≥ 90 mmHg three or more consecutive times. Information regarding other disease, family history, and prior therapy of every patient was collected from self-reported medical history.

### Laboratory analysis

Blood samples were obtained from each patient from the cubital vein after at least 12 h of fasting. Concentrations of

total cholesterol (TC), triglyceride (TG), low-density lipoprotein-cholesterol (LDL-C), and high-density lipoprotein-cholesterol (HDL-C) were measured using an automatic biochemistry analyzer (7150; Hitachi, Tokyo, Japan) in an enzymatic assay. Lp(a) was determined by immunoturbidimetry method [LASAY Lp(a) auto; SHIMA Laboratories Co., Ltd] with a normal value of < 30 mg/dL. An Lp(a) protein validated standard was used to calibrate the examination, and the coefficient of variation value of repetitive measurements was < 10%. Concentrations of Fib were measured using a Stago auto analyzer by the Clauss method with an STA Fibrinogen kit (Diagnostica Stago, Taverny, France).

### Statistical analysis

The values were expressed as the mean ± SD or median (25–75th percentile) for the continuous variables and the number (percentage) for the categorical variables. The Kolmogorov–Smirnov

test was used to test the distribution pattern. The differences in clinical characteristics between groups were analyzed using Student t test, Mann–Whitney U test, *χ*^2^ tests, or Fisher exact test when appropriate. The event-free survival rates among groups were estimated by the Kaplan–Meier method and compared by the log-rank test. Univariate and multivariate Cox regression analyses were performed to calculate the hazard ratios (HRs). A P value of less than 0.05 was considered statistically significant. The statistical analyses were performed with SPSS, version 22.0, software (SPSS, Chicago, IL) and R language, version 3.5.2 (Feather Spray).

## Results

### Baseline characteristics

The baseline characteristics of the study participants were shown in Table 1. Over a median of 37.1 months (25–75th percentile 22.5–55.4 months) follow-up period, 395 CVEs occurred (160 died, 78 suffered non-fatal MI, and 157 had strokes). Patients suffered CVEs tended to be older (*p* < 0.001), with higher prevalence of hypertension (*p* = 0.005), DM (*p* < 0.001), and lower BMI (*p* = 0.016). There was no significant difference regarding the baseline lipid profiles (TG, TC, LDL-C, HDL-C, apoA1, apoB, all *p* > 0.05) except Lp(a) levels (p = 0.001). Significantly, the concentration of Fib and D-dimer were higher in patients with CVEs (all *p* < 0.05). Meanwhile, the rate of statin usage was lower (*p* = 0.005) at admission while balanced (p > 0.05) at discharge in CVEs compared with patients without events. However, The HR of baseline characteristics with future CVEs were presented in Additional file 1. Table S1.

**Table 1:** Baseline characteristics of study patients

	Total	Events	No events	*p* value
	n = 8417	n = 395	n = 8022	
Clinical characteristics				
Age, years	57.4 ± 10.8	62.2 ± 10.2	57.2 ± 10.8	< 0.001
Male sex, (%)	71.7	71.6	71.7	0.993
BMI (kg/m^2^)	25.8 ± 3.2	25.5 ± 3.2	25.9 ± 3.2	0.016
Hypertension, (%)	62.0	68.8	61.7	0.005
Dyslipidemia, (%)	74.8	72.3	74.9	0.259
Diabetes Mellitus, (%)	27.5	37.1	27.0	< 0.001
Family history of CAD, (%)	13.6	14.3	13.5	0.097
Current smoker, (%)	54.4	54.3	54.4	0.968
Laboratory findings				
TC (mmol/L)	4.16 ± 1.17	4.17 ± 1.26	4.15 ± 1.17	0.819
LDL-C (mmol/L)	2.53 ± 1.01	2.53 ± 1.11	2.53 ± 1.00	0.970
HDL-C (mmol/L)	1.06 ± 0.29	1.05 ± 0.29	1.06 ± 0.29	0.662
TG (mmol/L)	1.50 (1.10–2.10)	1.48 (1.06–2.10)	1.50 (1.10–2.10)	0.538
Lipoprotein(a) (mg/dL)	15.18 (6.74–36.79)	19.24(9.01–45.58)	15.00 (6.66–36.26)	0.001
apoA1 (g/L)	1.33 ± 0.29	1.34 ± 0.30	1.33 ± 0.29	0.726
apoB (g/L)	0.92 ± 0.30	0.93 ± 0.31	0.92 ± 0.30	0.609
Fibrinogen(g/L)	3.24 ± 0.79	3.35 ± 0.81	3.23 ± 0.78	0.003
D-dimer (ug/mL)	0.42 ± 0.62	0.55 ± 0.66	0.42 ± 0.62	< 0.001
Medications at admission				
Statins, (%)	75.5	68.3	75.8	0.005
Aspirin, (%)	83.6	82.3	83.6	0.557
ACEI, (%)	12.5	13.3	12.5	0.682
ARB, (%)	12.8	10.6	12.9	0.361
β-blockers, (%)	48.2	48.7	48.1	0.893
CCB, (%)	19.2	15.9	19.3	0.228
Medications at discharge				
Statins, (%)	94.0	95.5	93.9	0.313
Aspirin, (%)	96.2	96.9	96.2	0.638
ACEI, (%)	22.2	26.3	22.0	0.096
ARB, (%)	23.0	26.3	22.9	0.175
β-blockers, (%)	77.9	80.6	77.8	0.278
CCB, (%)	38.1	35.3	38.2	0.323

Data are expressed as mean ± SD or median (25–75th percentile) unless otherwise indicated. *ACEIs* ACE inhibitors; *ARBs* angiotensin receptor blockers; *CCB* calcium channel blocker

### Association of plasma Lp(a) Levels and CVEs

**Fig. 1:**
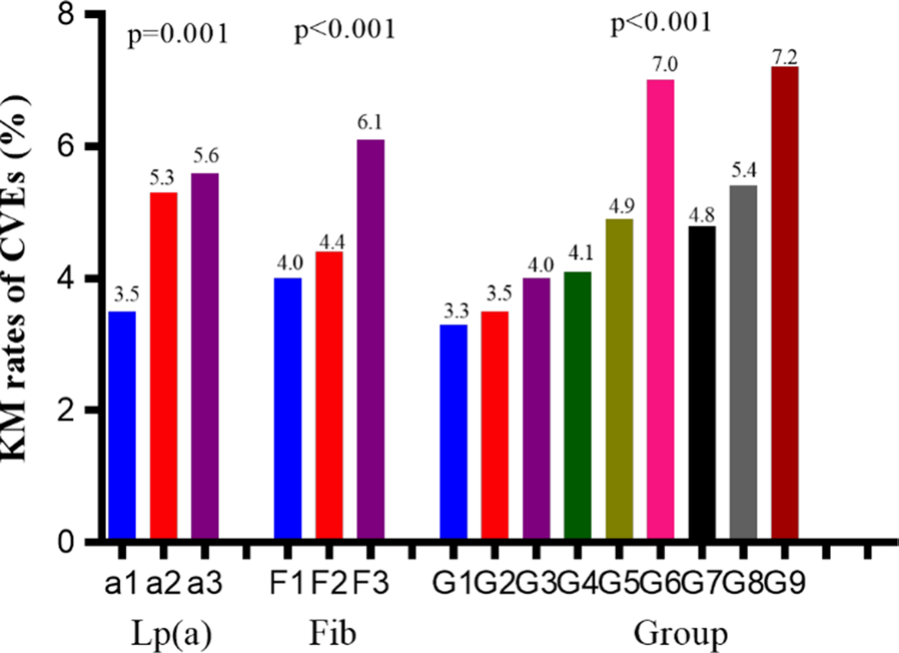
The KM rates of CVEs in Fib, Lp(a), and combined groups

**Fig. 2:**
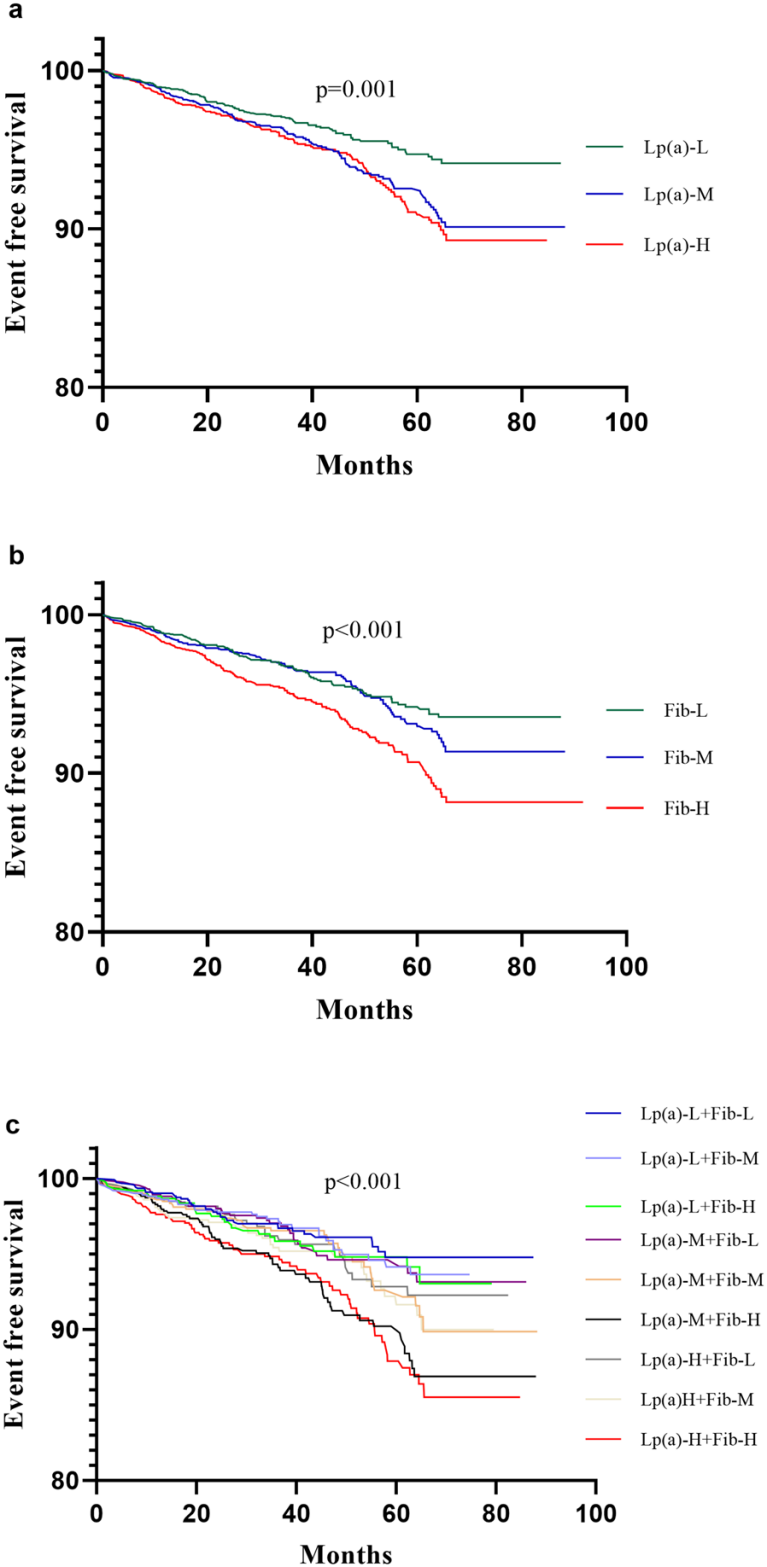
The event-free survival rate in Fib, Lp(a), and combined groups

In the current analysis, the subjects were assigned to 3 groups according to Lp(a) levels (Lp(a)-L: < 10 mg/dL, Lp(a)-M:10–29.9 mg/dL, Lp(a)-H ≥ 30 mg/dL). As shown in Fig. 1, the prevalence of CVEs in the Lp(a)-L, Lp(a)-M, and Lp(a)-H groups was 3.5%, 5.3%, and 5.6%, respectively (p < 0.001). Kaplan–Meier analysis (Fig. 2a) showed that Lp(a)-H subjects had the lowest event-free survival rate among the three groups (*p* = 0.001). As presented in Table 2, univariate Cox regression models showed that Lp(a)-M, and Lp(a)-H group had 1.468-fold and 1.580-fold higher risk of CVEs compared with Lp(a)-L group [Lp(a)-M: HR (95% CI) 1.468 (1.142–1.886), *p* = 0.003; Lp(a)-H: HR (95% CI) 1.580 (1.227–2.033), *p* < 0.001]. Additional adjustment for other variables in the multivariate Cox regression models did not change the significance of the association [Lp(a)-M: HR (95% CI) 1.531 (1.128–2.079), *p* = 0.006; Lp(a)-H: HR (95% CI) 1.786 (1.315–2.426), *p* < 0.001; Table 3].

**Table 2:** Association of fibrinogen and Lp(a) categories with clinical outcomes

Risk factor	Tertile/range	KM rates(%)	Hazard ratio	(95% CI)	*p* value
Lp(a) categories	Total (mg/dL)				< 0.001
	Lp(a)-L (< 10)	3.5	Reference		
	Lp(a)-M (10–29.9)	5.3	1.468	1.142–1.886	0.003
	Lp(a)-H (≥ 30)	5.6	1.580	1.227–2.033	< 0.001
Fibrinogen categories	Total (g/L)				< 0.001
	Fib-L(< 2.84)	4.0	Reference		
	Fib-M(2.85–3.42)	4.4	1.123	0.867–1.455	0.380
	Fib-H(≥ 3.43)	6.1	1.631	1.282–2.074	< 0.001
Combined categories	Total				< 0.001
	G1(Lp(a)-L + Fib-L)	3.3	Reference		
	G2(Lp(a)-L + Fib-M)	3.5	1.091	0.697–1.707	0.704
	G3(Lp(a)-L + Fib-H)	4.0	1.234	0.771–1.977	0.381
	G4(Lp(a)-M + Fib-L)	4.1	1.164	0.741–1.828	0.509
	G5(Lp(a)-M + Fib-M)	4.9	1.406	0.914–2.162	0.121
	G6(Lp(a)-M + Fib-H)	7.0	2.135	1.446–3.152	< 0.001
	G7(Lp(a)-H + Fib-L)	4.8	1.348	0.849–2.140	0.206
	G8(Lp(a)-H + Fib-M)	5.4	1.578	1.026–2.426	0.038
	G9(Lp(a)-H + Fib-H)	7.2	2.215	1.506–3.257	< 0.001

Data are expressed as HR (95% CI). *L* low, *M* medium, *H* high

**Table 3:** Adjusted association of fibrinogen and Lp(a) categories with clinical outcomes

Risk factor	Tertile/range	KM rates (%)	Hazard ratio	(95% CI)	*p* value
Lp(a) categories	Total				0.001
	Lp(a)-L (< 10)	3.5	Reference		
	Lp(a)-M (10–29.9)	5.3	1.531	1.128–2.079	0.006
	Lp(a)-H (≥ 30)	5.6	1.786	1.315–2.426	< 0.001
Fibrinogen categories	Total				0.002
	Fib-L(< 2.84)	4.0	Reference		
	Fib-M(2.85–3.42)	4.4	1.001	0.726–1.379	0.996
	Fib-H(≥ 3.43)	6.1	1.558	1.162–2.089	0.003
Combined categories	Total				0.002
	G1(Lp(a)-L + Fib-L)	3.3	Reference		
	G2(Lp(a)-L + Fib-M)	3.5	1.203	0.687–2.107	0.518
	G3(Lp(a)-L + Fib-H)	4.0	1.476	0.831–2.619	0.184
	G4(Lp(a)-M + Fib-L)	4.1	1.482	0.846–2.596	0.169
	G5(Lp(a)-M + Fib-M)	4.9	1.511	0.866–2.636	0.146
	G6(Lp(a)-M + Fib-H)	7.0	2.307	1.409–3.777	0.001
	G7(Lp(a)-H + Fib-L)	4.8	1.912	1.085–3.369	0.025
	G8(Lp(a)-H + Fib-M)	5.4	1.707	0.984–2.962	0.057
	G9(Lp(a)-H + Fib-H)	7.2	2.656	1.628–4.333	< 0.001

Data are expressed as HR (95% CI). *L* low, *M* medium, *H* high. Covariates used for adjustment are age, sex, BMI, diabetes mellitus, hypertension, dyslipidemia, family history of CAD, active smoking, D-dimer, and statin treatment

### Association of plasma Fib Levels and CVEs

Similarly, patients were divided into 3 groups according to Fib levels (Fib-L: < 2.84 g/L, Fib-M:2.85–3.42 g/L, Fib-H: ≥ 3.43 g/L). The prevalence of CVEs in the Fib-L, Fib-M, and Fib-H groups was 4.0%, 4.4%, and 6.1%, respectively (*p* < 0.001). The event-free survival rate was lowest in the Fib-H group (*p* < 0.001, Fig. 2b). Compared to Fib-L group, the Fib-H group had 1.631-fold higher risk of CVEs [HR (95% CI) 1.631 (1.282–2.074), *p* < 0.001] even after adjusting for potential confounders [HR (95% CI) 1.558 (1.162–2.089), *p* = 0.003].

### Inter-relationship of Lp(a), Fib Levels and CVEs

To evaluate an interaction between plasma Lp(a) and Fib levels on the risk of CVEs, the subjects were assigned to 9 groups according to Lp(a) and Fib levels (G1(Lp(a)-L + Fib-L, G2(Lp(a)-L + Fib-M, G3(Lp(a)-L + Fib-H, G4(Lp(a)-M + Fib-L, G5(Lp(a)-M + Fib-M, G6(Lp(a)-M + Fib-H, G7(Lp(a)-H + Fib-L, G8(Lp(a)-H + Fib-M, G9(Lp(a)-H + Fib-H).

The occurrence of CVEs in the 9 groups was 3.3%, 3.5%, 4.0%, 4.1%, 4.9%, 7.0%, 4.8%, 5.4%, and 7.2%, respectively (*p* < 0.001, Fig. 1).  As shown in Fig. 2c, the event-free survival rate was lowest in the 6th and 9th group (p < 0.001). Hazard ratios were calculated for each group using the G1 (group 1, Lp(a)-L and Fib-L) as a reference (Table 2). After adjusting for potential confoundings, the 6th group (Lp(a)-M and Fib-H) and 9th group (Lp(a)-H and Fib-H) had 2.307-fold and 2.656-fold higher risk of CVEs [HR (95% CI) 2.307 (1.409–3.777), *p* = 0.001; 2.656 (1.628–4.333), *p* < 0.001, respectively, Table 3).

In the original model, the C-statistic values were 0.633 (95% CI 0.603–0.664) with traditional risk factors, (Table 4). Addition of Lp(a) categories to the original model induced slightly improvement in C-statistic [ΔC-statistic 0.010 (− 0.001–0.023), *p* = 0.088] but did not reach statistical significance. When added Fib categories to the original model did not improve the C-statistic [ΔC-statistic 0.003 (− 0.005–0.012), *p* = 0.443]. Nonetheless, the combined Lp(a) and Fib categories resulted in a slightly improvement in C-statistic [ΔC-statistic 0.013 (0.002–0.027), *p* = 0.033].

**Table 4:** C-statistic of Lp(a) and Fib categories for predicting CVEs

Models	C-statistic (95% CI)	ΔC-statistic (95% CI)	*p* value
Original model	0.633 (0.603-0.664)	–	–
Original model + Lp(a) categories	0.643 (0.612–0.674)	0.010 (− 0.001–0.023)	0.088
Original model + Fib categories	0.637 (0.606–0.668)	0.003 (− 0.005–0.012)	0.443
Original model + combined categories	0.647 (0.616–0.678)	0.013 (0.002–0.027)	0.033

Original model included traditional risk factors as age, sex, BMI, diabetes mellitus, hypertension, dyslipidemia, family history of CAD, active smoking

## Discussion

In this prospective, large-cohort study, we investigated the association of plasma Lp(a) and Fib on the prediction of CVEs in angiography-proven stable CAD patients. Our data clearly found that both Lp(a) and Fib were independent predictors of CVEs in patients with stable CAD. More interestingly, the study firstly indicated that the combined Lp(a) and Fib categories enhanced the predicting values by incrementally increasing risk of CVEs in this population. The adjusted HR for CVEs was 2.656-fold and 2.307-fold higher among stable CAD patients in the Fib-H with Lp(a)-H or Lp(a)-M group, respectively. Finally, adding Lp(a) and Fib to the Cox model increased the C-statistic by 0.013 beyond that achieved with any single biomarker. These findings suggested that the combination of Lp(a), a complex marker of cholesterol and anti-fibrinolysis, and Fib, a marker of coagulation state, could enhance the predictive value, which would help the future risk stratification of stable CAD patients.

It is uncertain whether plasma Lp(a) levels are associated with CVEs in patients with stable CAD although several studies have suggested an association of elevated Lp(a) concentrations with the risk of CVD including the primary prevention population, familial hypercholesterolemia, statin-treated patients, and so forth. Concerning the secondary prevention setting, especially in patients with stable CAD, the results were controversial due to unknown causes. The Copenhagen City Heart Study showed that for patients with Lp(a) concentrations between 30 and 76 mg/dL, 77 and 117 mg/dL, and above 117 mg/dL, the risk of MI increased by a 1.6-fold, 1.9-fold, and 2.6-fold compared with those below 5 mg/dL in the primary prevention setting [4]. Data in patients with FH showed that the high cardiovascular risk in these patients is further increased by their unusual Lp(a) concentrations, which tend to be 2–3 fold higher than in the general population [14]. Of note, in the secondary prevention setting for patients with established CAD, inconsistent data were observed [15, 16]. Among 569 patients having undergone PCI and LDL-C levels were well-controlled (< 100 mg/dL), those with higher Lp(a) levels had significantly higher risk of MACEs compared to patients with lower Lp(a) levels, while elevated Lp(a) values were an independent predictor of mortality and recurrence of ACS [17]. Recently, our data proved that elevated Lp(a) levels were significantly associated with the risk of MACEs in patients with CAD combined with DM or pre-DM [18]. However, for patients with recent ACS who are treated with statins, Lp(a) concentration was not associated with MACEs [19]. Based on this situation, we consecutively enrolled 8,417 patients who had angiography-proven stable CAD and followed up for a median of 37.1 months. The data clearly showed that high Lp(a) (≥ 30 mg/dL) was resulted in 1.786-fold CVEs risk compared with low Lp(a) levels (< 10 mg/dL).

Next, previous including our studies supported the notion that Fib, a coagulation factor, is also a marker for risk of CVD [20, 21]. In this study, we re-examined the role of Fib in prediction of CVEs, and finally proved that high Fib was related to 1.631-fold higher risk of CVEs compared with the low Fib level. Till now, Lp(a) is thought to mediate clinical events by 3 main mechanisms, pro-atherogenic effects via its LDL-C moiety [3, 22], pro-inflammatory effects via its content of oxidized phospholipids [23] and anti-fibrinolytic effects via its apolipoprotein(a) component [24]. Lp(a) has high homology (75–99%) to plasminogen but lacks protease activity, and therefore has been hypothesized to inhibit fibrinolysis and mediate prothrombotic potential. Therefore, we hypothesize that there might be an enhanced impact of Lp(a) and Fib due to their pathophysiological action and previous evidence. A previous study indicated that high Fib associated with high Lp(a) levels significantly increased the risk of CAD [25], the study was designed for the primary prevention and was restricted by the male population. Hence, in this secondary prevention population, we divided our patients into 9 subgroups and found that the Fib-H with Lp(a)-H or Lp(a)-M group had 2.656-fold and 2.307-fold higher risk of CVEs, and the combination of Lp(a) and Fib categories improved the predictive value for CVEs beyond any biomarker alone.

Nevertheless, our study had several limitations. First of all, this is a study among Chinese population with stable CAD in the statin era, and whether the data applied to other populations need to be testified. Secondly, the Lp(a) and Fib concentrations were only measured at baseline, and the alterations of these biomarkers may also be clinically significant during the follow-up period. Finally, as this was an observational study, further investigations are needed to clarify the underlying mechanism of the associations.

## Conclusions

In conclusion, according to the functional similarity of Lp(a) and Fib in pro-atherogenic and anti-fibrinolytic effects, we examined the potential role of combining Lp(a) with Fib for predicting CVE in 8,417 patients with stable CAD and followed up an average of 37.1 months. Data firstly suggested that Lp(a) plus Fib could significantly enhanced predicting value for cardiovascular outcome in patients with stable CAD compared to that of Lp(a) or Fib alone.

## Supplementary information

**Additional file 1:** **Table S1.** Association of baseline clinical variables with CVEs.

## Data Availability

The datasets used and/or analyzed during the current study are available from the corresponding author on reasonable request.

## Abbreviations

CVD: Cardiovascular disease
CAD: Coronary artery disease
CVEs: Cardiovascular events
Lp[a]: Lipoprotein(a)
Apo(a): Apolipoprotein(a)
Fib: Fibrinogen
ACS: Acute coronary syndrome
MI: Myocardial infarction
TC: Total cholesterol
TG: Triglyceride
LDL-C: Low-density lipoprotein-cholesterol
HDL-C: High-density lipoprotein-cholesterol
