# Supplementary material for: Prognostic utility of lipoprotein(a) combined with fibrinogen in patients with stable coronary artery disease: a prospective, large cohort study
Source: J Transl Med. 2020 Oct 1;18:373. doi: 10.1186/s12967-020-02546-y (PMC7528376; doi:10.1186/s12967-020-02546-y)
Supplement: Supplementary file 1 — Additional file 1: Table S1. Association of baseline clinical variables with CVEs. [file 12967_2020_2546_MOESM1_ESM.docx]

**Supplementary Table 1**

| Variables | Hazard Ratio (95% CI) | p value |
| --- | --- | --- |
| Age, years | 1.045(1.034-1.055) | <0.001 |
| Male sex, (%) | 0.960(0.772-1.195) | 0.718 |
| BMI (kg/m^2^) | 0.965(0.934-0.997) | 0.030 |
| Hypertension, (%) | 1.336(1.080-1.653) | 0.008 |
| Dyslipidemia, (%) | 0.862(0.691-1.075) | 0.187 |
| Diabetes Mellitus, (%) | 1.554(1.267-1.906) | <0.001 |
| Family history of CAD, (%) | 0.935(0.703-1.244) | 0.646 |
| Current smoker, (%) | 0.991(0.812-1.208) | 0.926 |
| **Laboratory findings** |  |  |
| TC (mmol/L) | 1.009(0.926-1.100) | 0.839 |
| LDL-C (mmol/L) | 1.008(0.911-1.115) | 0.878 |
| HDL-C (mmol/L) | 0.922(0.645-1.317) | 0.655 |
| TG (mmol/L) | 0.973(0.893-1.060) | 0.535 |
| Lipoprotein(a) (mg/dL) | 1.005(1.002-1.009) | 0.003 |
| apoA1 (g/L) | 0.903(0.631-1.293) | 0.576 |
| apoB (g/L) | 1.015(0.732-1.409) | 0.927 |
| Fibrinogen(g/L) | 1.221(1.089-1.370) | 0.001 |
| D-dimer (ug/mL) | 1.151(1.077-1.231) | <0.001 |
| **Medications at admission** |  |  |
| Statins, (%) | 0.775(0.604-0.994) | 0.045 |
| Aspirin, (%) | 0.775(0.567-1.060) | 0.110 |
| ACEI, (%) | 0.885(0.602-1.299) | 0.533 |
| ARB, (%) | 1.249(0.818-1.906) | 0.304 |
| β-blockers, (%) | 1.004(0.775-1.301) | 0.974 |
| CCB, (%) | 1.245(0.872-1.778) | 0.227 |

**Association of baseline clinical variables with CVEs**

Data are expressed as HR (95%CI). ACEIs, ACE inhibitors; ARBs, angiotensin receptor blockers; CCB, calcium channel blocker.
